# Physical, Optical, and Mechanical Properties of Ceramic Materials after Coffee Immersion and Evaluation of Cleaning Impact with Different Oral Hygiene Tools

**DOI:** 10.3390/ijerph192215047

**Published:** 2022-11-15

**Authors:** Nasser M. Al Ahmari, Maram A. Alahmari, Mohammed M. Al Moaleem, Raghad S. A. Alshahrani, Fatimah F. Alqahtani, Waad Saeed Mohammed, Bandar M. A. Al-Makramani, Vini Mehta, Aida Meto, Agron Meto

**Affiliations:** 1 Prosthetic Department, College of Dentistry, King Khalid University, Abha 62529, Saudi Arabia; 2 Saudi Board Prosthodontic Resident, College of Dentistry, King Khalid University, Abha 62529, Saudi Arabia; 3 Department of Prosthetic Dental Science, College of Dentistry, Jazan University, Jazan 45142, Saudi Arabia; 4 Faculty of Dentistry, University of Ibn al-Nafis for Medical Sciences, Sana’a 4337, Yemen; 5 Dental Interns, College of Dentistry, King Khalid University, Abha 62529, Saudi Arabia; 6 Department of Public Health Dentistry, Dr. D.Y. Patil Dental College and Hospital, Dr. D.Y. Patil Vidyapeeth, Pune 411018, India; 7 Department of Dentistry, Faculty of Dental Sciences, University of Aldent, 1007 Tirana, Albania; 8 Clinical Microbiology, School of Dentistry, University of Modena and Reggio Emilia, 41125 Modena, Italy

**Keywords:** oral hygiene tools, coffee, ceramic, CAD/CAM, fracture type

## Abstract

This study aimed to evaluate the effect of three oral hygiene tools, a regular toothbrush, an electronic toothbrush, and mouthwash, on the color stability of three different computer-aided design (CAD) and computer-aided manufactured (CAM) ceramic blocks. Feldspathic ceramic (Vita Triluxe Forte), hybrid resin ceramic (Vita Enamic), and lithium disilicate glass-ceramic (IPS e.max CAD) were used in this study. A CAD/CAM system and 81 (27 of each material) samples of ceramic blocks were used. All samples were immersed in black coffee for 15 days, and the coffee was changed twice per day. Using a spectrophotometer probe, samples on a grey background were scanned, and physical properties like surface hardness and depth were measured using interferometry and a 3D non-contact surface metrology. After 30 days of application of oral hygiene tools, instruments were used to measure various physical, mechanical, and optical properties. Vita Triluxe had the highest average color variation values (Δ*E_00_*) after 15 and 30 days of immersion in coffee in both regular and electronic toothbrushes. Moreover, IPS e.max CAD had the least Δ*E_00_* values with no significant differences among the groups. The surface roughness (Ra) of the Vita Enamic ceramic increased when using a regular toothbrush, and the surface height (Rz) for the Vita Enamic ceramic increased when an electronic toothbrush was used. IPS e.max CAD had the greatest modules of elasticity forces, and Vita Triluxe Forte had the lowest when used with a regular toothbrush. The Δ*E_00_* values of the tested materials were minimally increased or decreased after 30 days of cleaning, and all were clinically acceptable. Ra and Rz were the highest for Vita Enamic in comparison to the other groups. The highest percentage in IPS e.max CAD was associated with a type 1 fracture, whereas type 3 was predominantly observed with Vita Enamic, and type 2 in the Vita Triluxe group without significant differences.

## 1. Introduction

Dental ceramics are materials that are a part of systems that are created for generating dental prostheses due to their aesthetics and biocompatibility [1], which are then used to replace missing or damaged dental structures [2]. There has been a surge in demand over the past 10 years for computer-aided design and computer-aided manufacturing (CAD/CAM) for improved aesthetics and color stability, leading to an increased quality of care and treatment [3]. Most widely known dental ceramics include feldspathic (Vita Triluxe Forte), hybrid resin ceramic (Vita Enamic), and lithium disilicate glass-ceramic (IPS e.max CAD). These materials are distinguished by strength, brittleness, transparency, and roughness. They are biocompatible, having minimal plaque adhesion and better color stability [4,5].

Feldspathic porcelains are widely employed ceramic materials by dental professionals to fabricate indirect restorations and veneers [6]. More recent machining systems have been developed, such as the Vita Triluxe Forte (a form of feldspathic glass ceramic) containing 30% fine-particle feldspar glass ceramic, which has improved strength [7,8]. Hybrid ceramics are characterized with high loading capacities having the flexibility of resin and the strength of ceramic due to the mix of ceramic and polymer [9]. The only hybrid ceramic up to this point is Vita Enamic [10]. Its unique microstructure, grain size, and grain distribution differentiate it from other ceramics [1,11]. Lithium disilicate glass ceramic (LDGC), which is usually marketed as IPS e.max CAD, is made up of a 70% crystalline matrix [12]. Additionally, well-documented characteristics of its distinctive microstructure are its biocompatibility, excellent light transmission, good mechanical strength, low thermal conductivity, and abrasion resistance [13,14].

The success and durability of dental restorations are significantly impacted by the color stability of the work. Color stability during surface processing is highly dependent on surface roughness and surface-free energy. Due to devices such as spectrophotometers and colorimeters, it is possible to evaluate the color stability of these ceramic materials both in vivo and in vitro [15]. A 3D color space called CIELAB was established by the Commission International De l’Eclairage (CIE) in 1976 as a representation for the perception of color stimuli, where *L stands for lightness, and *a and *b stand for chromaticity coordinates. The perception of tooth color is acknowledged to be an intricate phenomenon, and the visual assessment of tooth color may get affected by external and internal stains, tooth mineral content, lighting, color of the lip, and assessor experience. Numerous in vitro investigations indicate that ceramics have more superior color stability compared to other dental materials [13,16,17,18,19,20]. Alghazali et al. in 2019 evaluated the influence of Arabic coffee on the color of glazed or polished different porcelain veneers. A significant difference in the ΔE values before and after immersing in Arabic coffee for all materials used in their study. Furthermore, significant differences in color changes were noticed between the glazed and polished specimens [20].

Even though these materials can adequately replicate the dental structure, parameters, like surface roughness (Ra) and surface height (Rz), may be impacted by exposure to the hostile environment of the oral cavity [21]. The color and light reflection are negatively impacted by a rough surface. The lower the optical reflection, the rougher its surface [22]. Roughness caused by restoration adjustment may worsen surface imperfections and, as a result, weaken the porcelain [23,24]. Ra and Rz are directly linked with polished surfaces and the propagation of subcritical cracks [15,16,17,18,19,20,21,22,23,24,25]. Nutrition, salivary rate, temperature differential, and various bacterial flora in the oral cavity are some of the additional elements that may cause rough ceramic surfaces intraorally [17,21].

Personal oral hygiene involves removing bacteria plaque from teeth and gums to preserve oral health [26]. Toothbrushing alone has limited impact on dental caries as the main preventative effect is brought about by the regular administration of fluoride from the dentifrice. Electric toothbrushes are easy to use as the automatic oscillating and rotating movements require less effort from an individual [27]. To compensate for inadequacies in mechanical plaque reduction, mouthwashes like Listerine^®^ Zero™ are widely used in dentistry and are useful for reducing dental plaque accumulation, gingivitis, and halitosis. However, mouthwashes contain alcohol and hydrogen peroxide that reduce the microhardness of ceramic materials and ultimately lead to the softening and alteration of organic components of these materials rendering them more susceptible to erosion and discoloration [18,28].

According to previous data, mechanical and chemical plaque control agents might cause damage to surface and discoloration [29,30,31,32,33,34]. Derafshi, et al. examined the color alterations that occurred in monolithic zirconia and feldspathic porcelain when submerged in distilled water, Listerine^®^, and chlorhexidine (CHX). They discovered that both materials suffered from some color changes after being submerged in the Listerine^®^ and CHX mouthwashes. Additionally, they noticed that CHX had the greatest E values, followed by Listerine^®^, and pure water [35]. However, there are some reports of different types of mouthwash and regular or electronic toothbrushes, which have affected the mechanical and color stability of CAD/CAM ceramic materials with limited data on the effects of coffee immersion and its current developments in CAD/CAM materials [3,7,23].

Therefore, the aim of this in vitro study was to evaluate the effects of three different oral hygiene tools such as regular and electronic toothbrushes and mouthwash on the color stability of three different CAD/CAM materials. Feldspathic ceramic (Vita Triluxe Forte), hybrid resin ceramic (Vita Enamic), and lithium disilicate glass-ceramic (IPS e.max CAD) were assessed after immersion in coffee for 15 days. This study also aimed to measure average color changes (Δ*E_00_*), Ra, Rz, biaxial fracture, and modules of elasticity forces as well as the fracture types of ceramic materials.

## 2. Materials and Methods

### 2.1. Study Design and Sample Size Estimation

**Figure 1:**
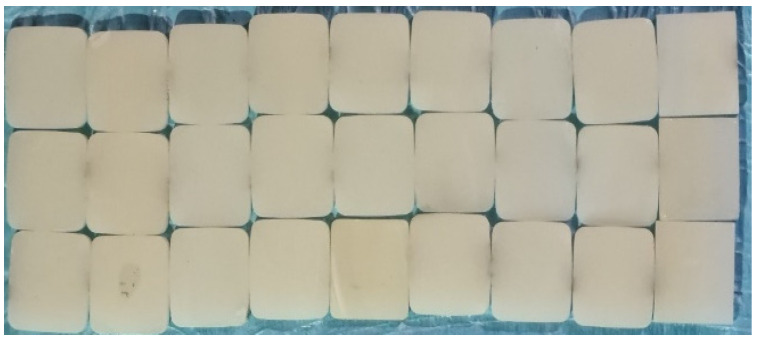
Samples mounted exposing the brushed surfaces.

This in vitro study evaluated three commercially accessible and commonly used CAD/CAM ceramic prosthetic materials to determine the effect of oral hygiene tools on Ra, Rz, Δ*E_00_* compressive fracture resistance, biaxial fracture, modules of elasticity, and fracture subtypes. Sample size was calculated by using G*Power software (latest ver. 3.1.9.7; Heinrich-Heine-Universität Düsseldorf, Düsseldorf, Germany). In this study, there were 3 groups. Assuming a moderate effect size between the groups for fracture resistance (0.25) at 5% level of significance and 85% power, the sample size is 27 subjects per group. Therefore, the total sample size required is 27 × 3 = 81 subjects (Figure 1) [36].

### 2.2. Construction, Grouping, and Surface Treatment of the Specimens

Feldspathic ceramic (Vita Triluxe Forte), hybrid resin ceramic (Vita Enamic), and lithium disilicate glass-ceramic (IPS e.max CAD) were used (Vita Zahnfabrik, H. Rauter Bad Säckingen, Germany). Using a CAD/CAM system (Amann Girrbach, GmbH, Durrenweg 40 75,177 Pforzheim, Deutchland), 81 (27 per group) samples of ceramic blocks were cut into rectangular slices with a thickness of 1.5 × 0.2 ± 0.2 mm and a dimension of 14 × 2 ± 0.2 mm. Thickness was measured using a digital caliper which was followed by glazing and sintering the samples for 2 h at 1550 °C in a dental ceramic furnace. Prior to immersion, ultrasonic scrubbing of ceramic materials was carried out with distilled water for 10 min to remove left out debris, with compressed air drying for 20 s. Based on the type of oral hygiene aids (regular toothbrush, electronic toothbrush, and mouthwash), samples for each material group were further divided into 3 equal subgroups of 9 each.

### 2.3. Coffee Immersion and First Color Measurements

All samples were immersed in black coffee (Starbucks, Washington, DC, USA) for 15 days, and the coffee was changed twice per day. Using a spectrophotometer probe (SCIEX, Framingham, MA, USA), samples on a grey background were scanned. One operator measured the color of each sample against a grey backdrop before exposing it to various oral hygiene products. The term “lightness” is denoted by the “L” value. The higher the “L” value, the greater the lightness. Red and green colors revealed positive and negative values and they are represented by the letter “a”, respectively. However, “b” means yellow and blue colors revealed positive and negative values, respectively. The CIELAB color system, which provided the mathematical values for the 3D color measurements, was used to record the parameters for each CAD/CAM ceramic material. Baseline color parameters (L1, a1, and b1) were measured three times for each specimen at the same time of day before using oral hygiene tools, and the average was utilized to represent various color coordinates [32,37,38,39,40,41].

### 2.4. Arithmetic Ra and Rz Measurements

Ra and Rz of samples were measured using interferometry and 3D non-contact surface metrology (Bruker Contour GTK, Bruker Nano Surfaces Division, Tucson, AZ, USA). An upright scan interferometry lens and a field of interpretation regression filter were used, as well as a software package that controls tool locations, statistical evaluation, and graphical output. Ra and Rz were measured twice across all samples at a two-time interval, at baseline and 28 days after the application of oral hygiene tools. Each sample was scanned three times, and then the mean was calculated to determine Ra and Rz values in micrometer [3,7].

### 2.5. Application of Oral Hygiene Tools

The three oral hygiene tools were utilized and applied as per manufacturer’s instructions. Both traditional and electronic toothbrushes were used twice daily for 60 s with toothpaste. Each 30 s manual brushing session required 0.25 g of toothpaste, which was diluted with water to a ratio of 1:3 (manual toothbrush: Oral-B Pro-Expert Pro-Flex, Soft Manual Toothbrush; electric toothbrush used: Oral-B Db4.010 Pro expert battery powered toothbrush; mouthwash used: Listerine^®^; dentifrice used: Signal^®^ toothpaste) [32,42].

The samples were brushed with toothbrush using a circular motion, then rinsed with running water. We adjusted the time of working on the samples; early morning and at the end of duties (every 12 h). Secondly, we put the samples over a balance, and used the brushes over the samples with a constant load (2 kg ± 0.2 kg) and at the same direction for 30 s twice. We fixed time, load, direction, and time of brushing. After the tooth-brushing procedure, artificial saliva was replaced and the samples were stored in the artificial saliva in a dark glass container at room temperature for the entire duration of the testing period.

Twice a day, the mouthwash was applied for 60 s each time. To mimic everyday use, each sample was exposed to 20 mL of mouthwash at 37 °C for 15 days. This is similar to using mouthwash for 1 year, twice daily for 30 s. Every 12 h, the mouthwash solution was changed.

### 2.6. Color Parameters Measurements after Oral Hygiene Application

After 30 days of application, the samples were rinsed with distilled water and patted dry with tissue paper. After that, the same instruments, as mentioned previously, were used to measure color parameters as post-brushing readings, which were recorded as L2, a2, and b2. The Δ*E_00_* values were calculated using the following formula: ∆*E_00_* = (∆L*)^2^ + (∆a*)^2^ + (∆b*)^2^) × 1/2, where ∆L* is difference of L*, ∆a* is the variation of a*, and ∆b* is the variation of b* [3,7,8,39,41].

### 2.7. Measuring the Biaxial Fracture Forces and Modulus of Elasticity Forces

Biaxial and modules of elasticity forces of the samples were assessed with a computer-controlled universal testing machine (Zwick Z010/TN2A, Ulm, Germany) at the across-head speed of 0.5 mm/minutes using a rod with 4 mm diameter as suggested in ISO standard 6872 and in air at room temperature by using a universal mechanical testing apparatus using a 3 mm radius stainless steel hemispherical tip will be applied to the center of each sample. Biaxial and modules of elasticity forces were recorded automatically in Newton in MPa [43,44].

### 2.8. Failure Type Definition Assessment

**Figure 2:**
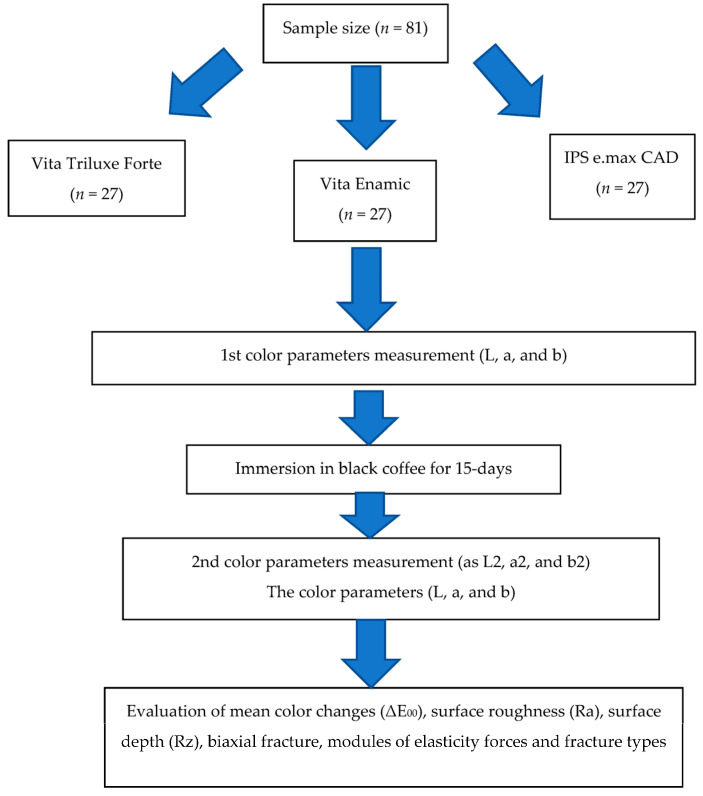
Representation of the groups and subgroups’ distribution for the various tests conducted in this study.

Each sample’s shape, the quantity of fragments that resulted from fractures, and the proportion of each type were noted. The following parameters served as the foundation for the classification of fracture types [25,36]. Type 1: a reparable specimen is split in half, with each half retaining 50% of the dimensions of the original shape. Type 2: a semi-reparable specimen can be divided into three to four parts, each of which is between 20% and 35% of the original size. Type 3: a non-reparable specimen that has been divided into at least four parts, each of which is less than 20% of the original specimen’s size [25,36]. Figure 2 shows the steps and type of the material groups and subgroups as well as the tests used, as mentioned previously.

### 2.9. Statistical Analysis

The data was entered and analysed using the Statistical Package for Social Sciences (SPSS) for Windows, Version 28.0. (IBM Corp, Armonk, NY, USA). Confidence intervals were set at 95%, and a *p*-value ≤ of 0.05 was considered statistically significant. The Δ*E_00_* values of ceramic materials were presented as Mean ± Std. Deviation (SD). After 15 days of black coffee immersion, then 30 days of cleaning, the Δ*E_00_* values of the Vita Triluxe Forte, Vita Enamic, and IPS e.max CAD blocks were determined. A One-Way ANOVA and a Mann—Whitney U test was used to compare mean/distributions of parameters over groups. A Pairwise Bonferroni test was used as post-hoc analysis.

## 3. Results

The Δ*E_00_* values of all the ceramic materials after immersion in coffee for 15 days or at baseline and after 30 days of application of different cleaning aids are summarized in Table 1. The Δ*E_00_* values of the tested materials minimally increased or decreased after 30 days of cleaning with a regular toothbrush, an electronic toothbrush, and mouthwash and all were clinically acceptable. The Δ*E_00_* values after coffee immersion for 15 days were found to be highest in Vita Triluxe ceramics after coffee immersions (2.62). Furthermore, the mean color change (Δ*E_00_*) after oral hygiene aids’ immersion for 30 days were found to be highest in both regular toothbrush and signal toothpaste and Listerine mouthwash in Vita Triluxe ceramics (2.64). In addition, the least Δ*E_00_* values after coffee immersion were found to be 1.19 and 1.28, respectively, in IPS e.max CAD after the 15th and 30th days of coffee immersion and oral hygiene application, respectively. No significant differences were seen in the Δ*E_00_* values at baseline and after 30 days cleaning amongst the groups and CAD/CAM materials.

Comparing the groups, it was revealed that Ra (0.56) and Rz (0.53) were the highest for the Vita Enamic material. The Ra of the Vita Enamic ceramic increased when using the regular toothbrush and Rz for Vita Enamic ceramic increased when the electronic toothbrush was used. No significant differences were seen in Ra and Rz amongst different ceramic materials (Table 2).

**Figure 3:**
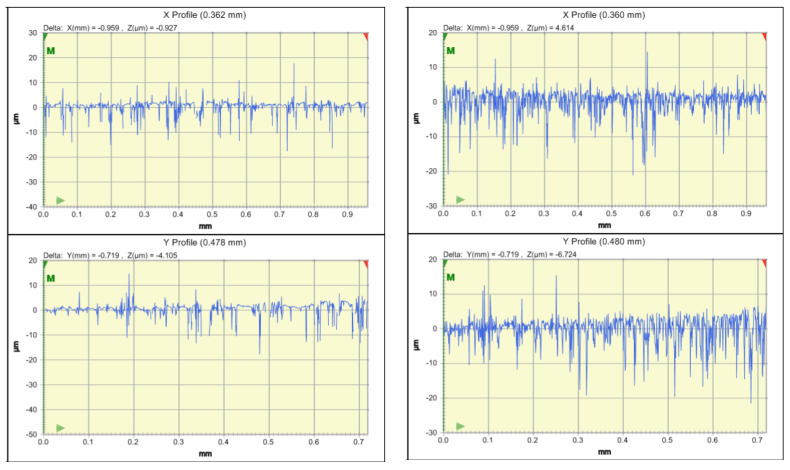

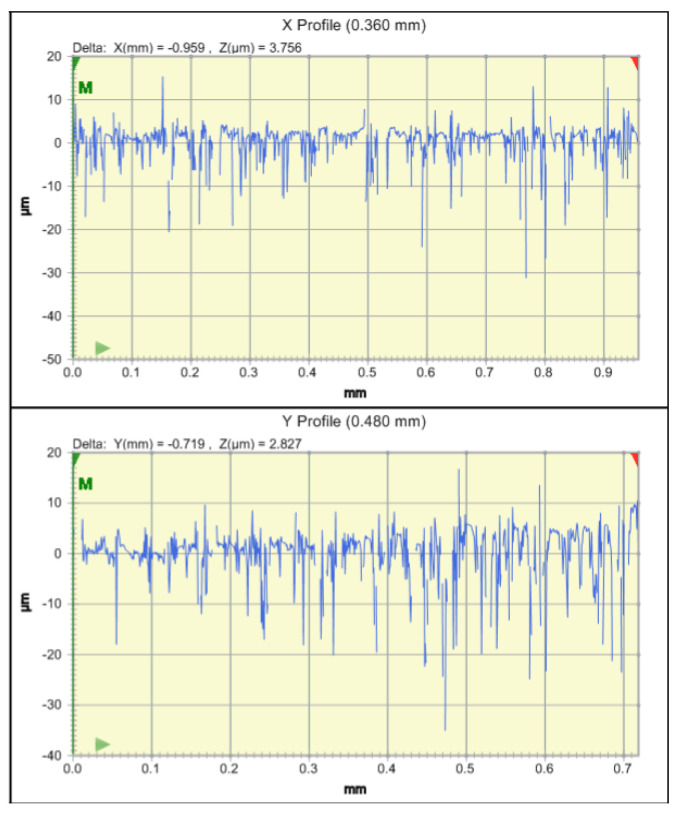
Ra and Rz amongst different ceramic materials.

Figure 3 shows a spectrophotograph depicting Ra and Rz of different ceramic materials which indicated that the Vita Enamic material had the highest Ra and Rz. Table 3 revealed that IPS e.max CAD had the highest mean biaxial fracture force resistance when used with the mouthwash and overall oral hygiene tools (100.26), whereas Vita Triluxe Forte had the lowest mean biaxial fracture force resistance when used with an electronic toothbrush and toothpaste (42.08). Thus, the overall fracture resistance (MPa) was highest for the IPS e.max ceramic group (96.10) and the lowest fracture resistance was seen for Vita Triluxe Forte. (43.74) Significant differences were also seen between the Vita Triluxe Forte and IPS e.max ceramic groups with different oral hygiene aids (Table 3).

**Figure 4:**
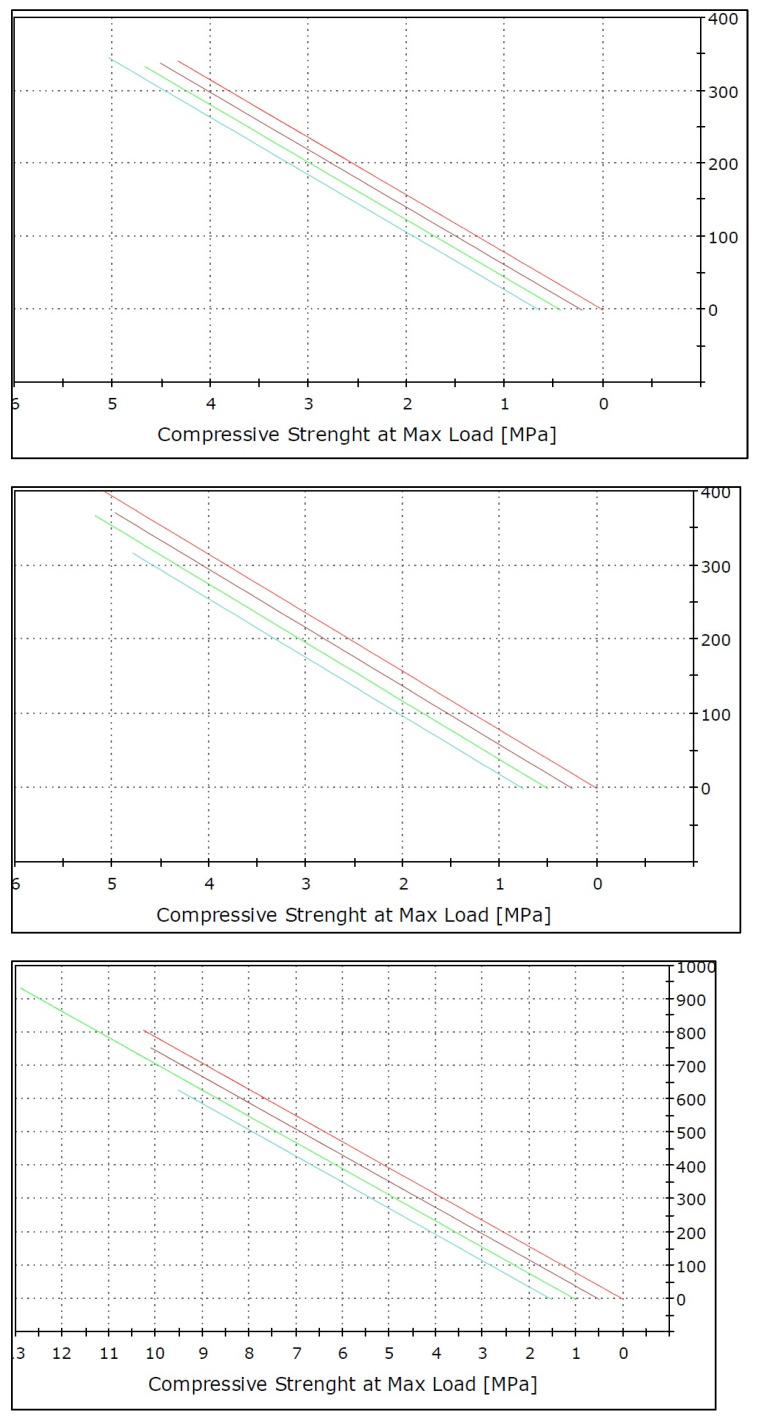
Mean biaxial fracture force resistance amongst different ceramic materials.

Table 4 revealed that IPS e.max CAD had the greatest modules of elasticity forces when used with a regular toothbrush and overall oral hygiene tools (56.32), whereas Vita Triluxe Forte had the lowest modules of elasticity forces when used with a regular toothbrush (39.16). Thus, the overall fracture resistance (MPa) was highest for the IPS e.max ceramic group (52.23) and the lowest fracture resistance was seen for Vita Triluxe Forte. (41.24) Significant differences were also seen between Vita Triluxe Forte and IPS e.max ceramic groups with different oral hygiene aids (Figure 4).

**Figure 5:**
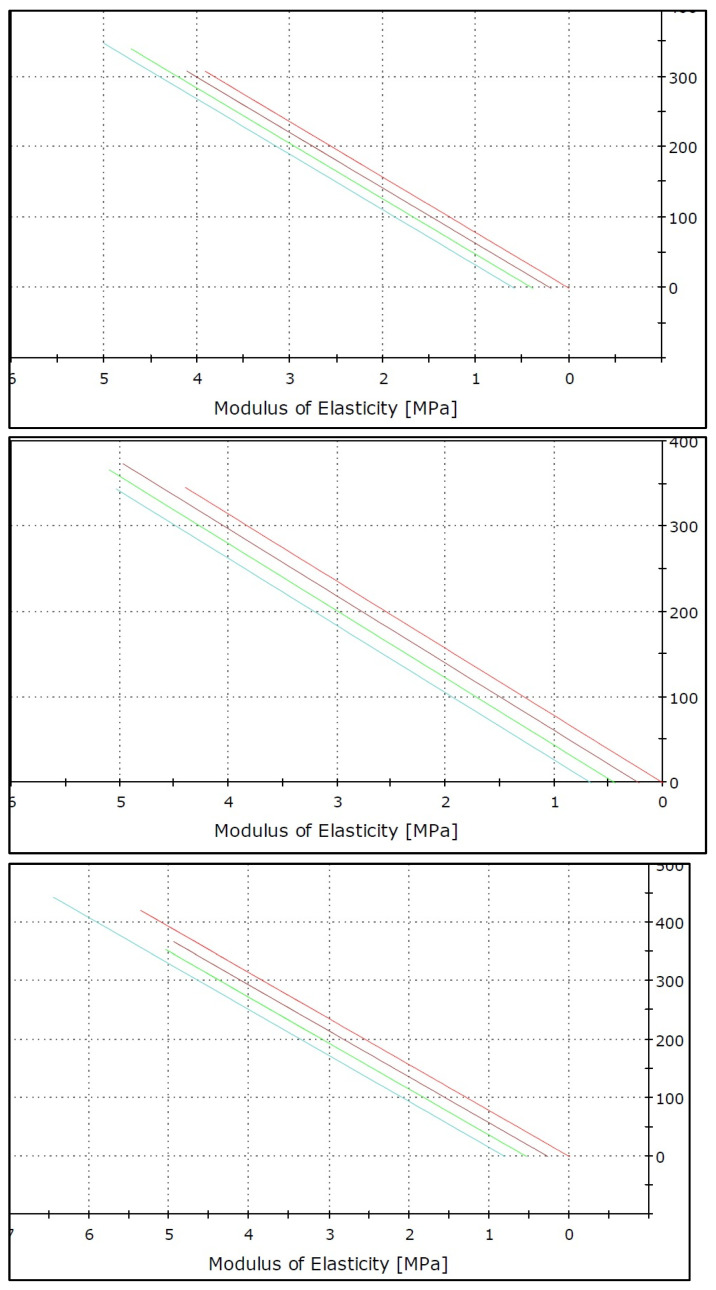
Modules of elasticity forces by ANOVA test among different ceramic materials.

Figure 5 presents the values of elasticity forces in MPa and Newtons in the *X*- and *Y*-axes. Significant differences were also seen between the IPS e.max ceramic group with different oral hygiene aids (Figure 5).

**Figure 6:**
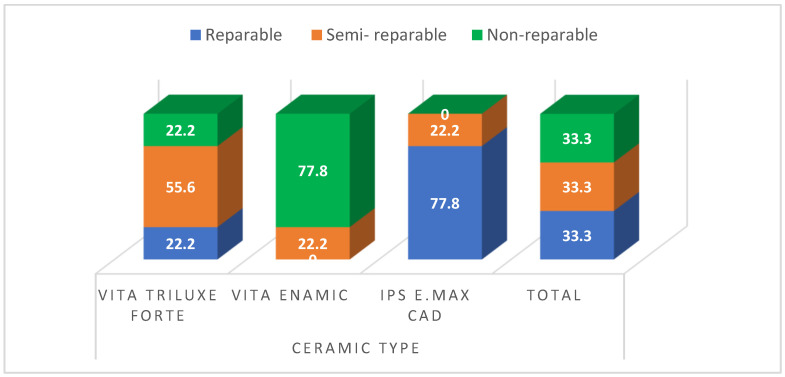
Fracture type percentage among different ceramic materials.

Table 5 revealed that there are significant differences amongst IPS e.max CAD with different oral hygiene tools. The highest percentage in IPS e.max CAD was associated with a type 1 fracture (reparable) (77.8). However, type 3 (non-reparable) was predominant with Vita Enamic (77.8), and type 2 (semi-reparable) with the Vita Triluxe group (55.6) without any significant differences. Figure 6 revealed that IPS e.max CAD was associated with a type 1 fracture (reparable), whereas type 2 (semi-reparable) and type 3 (non-reparable) were observed with Vita Triluxe Forte and Vita Enamic ceramic material groups without any significant differences.

## 4. Discussion

To our knowledge, all the previous studies measured the Δ*E_00,_* Ra, and Rz for ceramics after immersion in coffee and compared the effect of different oral hygiene tools in the stainability of the same materials.

Surface roughness plays a major role in determining ceramic strength. According to previous studies, increasing surface roughness can lead to increased bacterial plaque accumulation and gingival irritation [8,45]. When comparing the groups in our study, it was found that Ra and Rz were the highest for the Vita Enamic material. A study conducted on Ra of ceramics stated that only IPS e.max CAD showed no statistically significant increase in Ra in comparison with other ceramics [46]. After toothbrush abrasion, both ceramic polymers examined by Koizumi et al. revealed substantially rougher surfaces than feldspathic porcelain [47]. Our results were contrary to these findings as Ra for Vita Enamic ceramic increased with a regular toothbrush with dentifrice and Rz for Vita Enamic increased with an electronic toothbrush with dentifrice.

Different solutions can modify optical characteristics, surface roughness and color stability of ceramic materials [24,48]. Various in vitro trials have shown that ceramics conducted demonstrated superior ceramic quality [29]. Additionally, color stability may also be widely diminished due to use of various chemical and mechanical plaque control agents [35]. Mouthrinses have been advised as a supplementary treatment for antimicrobial control as they are effective at reducing dental plaque, gingivitis, and halitosis. They are typically composed of water, antimicrobial agents, salts, and alcohol; the concentrations of these substances can affect the mouthwashes’ pH. As mouthwashes decrease the oral pH, the sorption and solubility of ceramic materials increase, thereby leading to surface degradation and softening. Mouthwashes cause more surface roughness (Ra) of ceramics compared to other oral hygiene aids by diminishing the microhardness, making the material more flexible and vulnerable to erosion and discoloration [18]. Laborie at al conducted a systematic review in 2022 that stated that Listerine increased the roughness of ceramics due to the higher ethanol content causing low pH (4.1) and hydrolysis [4]. This hydrolysis may result in surface erosion and dissolution, which has a negative impact on wear, hardness, and surface integrity by softening the matrix and causing structural ion loss [18].

In our study, we used a Listerine-based mouthwash, which is routinely used to treat oral disorders. Significant discolorations were observed using the mouthwash after 7 and 15 days of immersion. The mean color change after 30 days of coffee immersion was found to be greatest in the regular toothbrush and Signal toothpaste and the Listerine mouthwash. These findings were consistent with previous research analyzing the discoloration effect of several mouthrinses [35,36,37,38,39,40,41,42,43,44,45,46,47,48,49]. Derafshi et al. investigated the discoloration of monolithic zirconia and feldspathic ceramics in two mouthrinses (Listerine and CHX). He discovered substantial color changes for zirconia samples after immersion in chlorhexidine and Listerine mouthwashes after 7 days of immersion [35]. Our results were contrary to the findings of Zajkani et al., wherein he investigated the effect of 0.2% CHX and post-immersion polishing on the color stability of composite resins and discovered that CHX-induced discoloration on the restorative material. Despite the fact that different restorative materials were used in the current study, similar results were found [50].

Coffee is one of the most widely drank beverages, not only in Saudi Arabia but also globally. With its increased consumption, cosmetic restorative procedures are critical for preserving the maximum amount of original teeth structure quality [51]. This study used coffee as a staining solution as it contained a copious number of chromogenic substances like tannin and chlorogenic acid compared to other beverages. Furthermore, its pH ranged from 4.9 to 5.2, which contributed to the discoloration. Coffee may stain all-ceramic materials and can be used to evaluate a discoloration potential of a material [20,52].

In the current study, the ceramic materials were soaked in coffee for two weeks which was congruent to the Al-Ahmari study of 2022, which used a similar immersion method and was contrary to the research conducted by Quek et al. and Saglam G et al., who immersed samples in the coffee solution for one week [18,53]. Haralur et al. [41] discovered DE00 values after staining lithium disilicate in coffee, tea, and chlorhexidine gluconate, showing the highest deterioration in lithium disilicate ceramics due to coffee. In a study published this year by Yerliyurt K et al., the maximum staining was detected after 28 days for Enamic restorations stored in coffee-wine across all groups [22]. These findings were contrary to our study, where we discovered that the mean color change after 14 days of coffee immersion was greatest in Vita Triluxe ceramics and when using a regular toothbrush and the Signal toothpaste. Our findings contradicted the previous findings, since the most discoloration was found with feldspathic ceramics, specifically Vita Trilux ceramics as they contain a maximum number of inorganic components. Ceramics are non-reactive, but their organic structure is fragile.

In a clinical scenario, a restoration that develops severe color degradation is regarded as a failure and it becomes important to measure these relative color changes. Several research reports have assessed color change using the CIELab system [32,35,41]. Our study employed a spectrophotometer due to its better precision, affordability, reproducibility, and flexibility to adapt data to numerous color measuring schemes or classifications. To further analyse the samples, a grey background was utilized. This was contrary to the Avram and Al-Ahmari et al. studies as they analysed color changes against a white and black background. It has been stated that a color change is visible to the naked eye when ∆E is greater than 1 [51,54].

In the present study, IPS e.max CAD, when used with mouthwash and other oral hygiene products, had the maximum mean biaxial fracture force resistance, whereas Vita Triluxe Forte had the lowest mean biaxial fracture force resistance when used with an electric toothbrush and toothpaste. Thus, the IPS e.max ceramic group had maximum fracture resistance, while Vita Triluxe Forte had the lowest. Significant variations were also observed in the IPS e.max ceramic group when using various dental hygiene products. Our study results were contrary to the study of Yilmaz and Okutan et al., who evaluated biaxial flexure strengths of monolithic zirconia specimens with various surface treatments; they published similar biaxial forces. After sintering, they concluded that the air abrasion group had the biaxial flexural strength with the highest average mean and standard deviation [55].

When used with a standard toothbrush, toothpaste, and other oral hygiene tools, IPS e.max CAD had the highest modules of elasticity forces, whereas Vita Triluxe Forte had the lowest modules of elasticity forces. Thus, the IPS e.max ceramic group had the highest overall fracture resistance, and Vita Triluxe Forte had the lowest. This was in contrast to the findings of Yoon et al. in 2018 wherein the evaluated CAD/CAM materials exhibited mostly catastrophic failures. Due to its greater elastic modulus compared to dentin, a ceramic prosthesis may absorb pressures to the tooth until catastrophic collapse occurs [8].

Fracture strength tests are typically used to assess various oral prosthesis materials behaviors under functional conditions. Longer storage times and mechanical testing should be carried out to better understand and analyze failure type. In our study, type 1 fracture was linked to IPS e.max CAD ceramics. Vita Enamic was the primary material used in the study by Alqahtani SM et al. in 2022, with lesser findings found in Vita Suprinity and Vitablocs Mark II samples and semi-reparable and non-reparable fracture modes [25]. These results were consistent with those of Al Moaleem et al., who investigated ceramics made of zirconia and feldspathic minerals [3,25]. However, this finding was contrary to our study as type 2 (semi-reparable) and type 3 (non-reparable) were observed with Vita Triluxe Forte and Vita Enamic ceramic material group without any significant differences.

A particular limitation of this study was its in vitro design. To assess long-term optical properties further, in vivo research is required to apply in clinical situations. In addition, it was discovered that the color stability of the restorative materials was altered by toothbrushing and salivary movements. We used artificial saliva for the entire duration of the testing period which is not the medium to check the optical properties which might be a disadvantage as it cannot reflect the exact properties of natural saliva. The measurements of color in this study were made against a grey background, but the results may have been different with other backgrounds. Further research to evaluate more optical features such as translucency and several undiscovered variables, such as compressive strength, hardness, and shear strength must be investigated to supplement the findings of the current study.

## 5. Conclusions

In light of this in vitro study and its limitations, the following conclusions were drawn:

1. The average color change (Δ*E_00_*) of the tested materials were minimally increased or decreased after 30 days of cleaning, and all were within the clinical acceptable values.
2. Ra and Rz were highest for Vita Enamic when comparing the groups. A significant difference between and within Vita Enamic group in both Ra and Rz among the different oral hygiene tools.
3. With mouthwash and overall, IPS e.max CAD had the highest mean biaxial and modulus of elasticity fracture force resistance, whereas Vita Triluxe Forte had the lowest with an electronic toothbrush and toothpaste. Significant differences were found for IPS e.max CAD with different oral hygiene tools.
4. The highest percentage of IPS e.max CAD was associated with the type 1 (reparable) fracture whereas the (non-reparable) type 3 was predominantly observed with Vita Enamic, and the (semi-reparable) type 2 in Vita Triluxe group without significant differences.

## Figures and Tables

**Table 1:** Mean color change (Δ*E_00_*) values at baseline and after 30 –days of cleaning with different oral hygiene tools and ceramic materials (*n* = 81).

Specimen Size	Δ*E_00_* after 15-Days Starbucks Coffee = Baseline before Oral Hygiene Tools Application	Type of Oral Hygiene Tools	Δ*E_00_* & SD after 30-Days of Oral Tools Applications	*p*-Value
9	2.62	Regular Toothbrush & Signal Toothpaste	Vita Triluxe 2.64 (1.10)	0.582
9	1.95		Vita Enamic 1.67 (0.19)	
9	1.21		IPS e.max CAD 1.32 (1.22)	
9	2.46	Electrical Toothbrush & Signal Toothpaste	Vita Triluxe 2.72 (2.01)	0.621
9	1.83		Vita Enamic 1.63 (1.20)	
9	1.19		IPS e.max CAD 1.28 (2.11)	
9	2.26	Listerine Mouthwash	Vita Triluxe 2.64 (0.30)	0.78
9	1.53		Vita Enamic 1.76 (0.92)	
9	1.22		IPS E.MAX CAD 1.18 (2.10)	

**Table 2:** Comparison of Ra and Rz amongst different ceramic materials.

Parameter	Type of Ceramic	Mean ± SD	Overall	*p*-Value
Ra for Vita Triluxe Forte	Regular toothbrush with toothpaste	0.14 (0.03)	0.14	0.121
	Electronic toothbrush with toothpaste	0.12 (0.09)		
	Mouthwash	0.16 (0.21)		
Ra for Vita Enamic	Regular toothbrush with toothpaste	0.60 (0.15)	0.56	0.081
	Electronic toothbrush with toothpaste	0.58 (0.14)		
	Mouthwash	0.49 (0.17)		
Ra for IPS E.max CAD	Regular toothbrush with toothpaste	0.21 (0.11)	0.20	0.224
	Electronic toothbrush with toothpaste	0.20 (0.10)		
	Mouthwash	0.18 (0.23)		
Rz for Vita Triluxe Forte	Regular toothbrush with toothpaste	0.22 (0.02)	0.23	0.521
	Electronic toothbrush with toothpaste	0.23 (0.09)		
	Mouthwash	0.24 (0.45)		
Rz for Vita Enamic	Regular toothbrush with toothpaste	0.47 (0.15)	0.53	0.632
	Electronic toothbrush with toothpaste	0.59 (0.19)		
	Mouthwash	0.53 (0.23)		
Rz for IPS E.max CAD	Regular toothbrush with toothpaste	0.19 (0.01)	0.32	0.088
	Electronic toothbrush with toothpaste	0.24 (0.23)		
	Mouthwash	0.26 (0.09)		

**Table 3:** Comparison of biaxial fracture forces amongst different ceramic materials.

Ceramic Type	Oral Hygiene Tools Type	Fracture Resistance (MPa) Mean (SD)	Overall Fracture Resistance (MPa)	*p*-Value
Vita Triluxe Forte	Regular toothbrush with toothpaste	43.85 (12.15)	43.74	0.073 *
	Electronic toothbrush with toothpaste	42.08 (44.48)		
	Mouthwash	43.42 (13.42)		
Vita Enamic	Regular toothbrush with toothpaste	50.79 (29.32)	48.19	0.420
	Electronic toothbrush with toothpaste	47.16 (25.30)		
	Mouthwash	46.63 (35.45)		
IPS e.max CAD	Regular toothbrush with toothpaste	98.29 (16.10)	96.10	0.076 *
	Electronic toothbrush with toothpaste	89.79 (11.53)		
	Mouthwash	100.26 (17.31)		

* Different superscript letters suggest statistically significant differences in each category (*p* < 0.05) as determined by ANOVA and Bonferroni tests.

**Table 4:** Comparison of modules of elasticity forces with different ceramic materials.

Ceramic Type	Oral Hygiene Tools Type	Fracture Resistance (MPa) Mean (SD)	Overall Fracture Resistance (MPa)	*p*-Value
Vita Triluxe Forte	Regular toothbrush with toothpaste	39.16 (11.12)	41.24	0.01 *
	Electronic toothbrush with toothpaste	43.16 (24.84)		
	Mouthwash	41.41 (15.24)		
Vita Enamic	Regular toothbrush with toothpaste	47.50 (23.12)	46.01	0.201
	Electronic toothbrush with toothpaste	46.55 (21.21)		
	Mouthwash	43.96 (18.34)		
IPS e.max CAD	Regular toothbrush with toothpaste	56.32 (16.10)	52.23	0.062 *
	Electronic toothbrush with toothpaste	53.63 (10.32)		
	Mouthwash	46.75 (25.21)		

* Different superscript letters suggest statistically significant differences in each category (*p* < 0.05) as determined by ANOVA and Bonferroni tests.

**Table 5:** Fracture type percentages among different ceramic materials.

Fracture Type		Reparable	Semi-Reparable	Non-Reparable
Ceramic type	Vita Triluxe Forte	22.2	55.6	22.2
	Vita Enamic	0.00	22.2	77.8
	IPS e.max CAD	77.8	22.2	0.00
	Total	33.3	33.3	33.3

## Data Availability

Not applicable.
